# Different metastasis promotive potency of small G-proteins RalA and RalB in *in vivo *hamster tumor model

**DOI:** 10.1186/1475-2867-11-22

**Published:** 2011-06-29

**Authors:** Vera A Rybko, Anna V Knizhnik, Andrei V Komelkov, Vasily N Aushev, Lyubov S Trukhanova, Elena M Tchevkina

**Affiliations:** 1 Department of Oncogenes Regulation, Institute of Carcinogenesis, Russian N.N. Blokhin Cancer Research Center, Kashirskoye shosse 24, 115478, Moscow, Russia

**Keywords:** metastasis, Ral proteins, invasion, Ral effector mutants, tumor growth

## Abstract

**Background:**

Previously we have shown that oncogenic Ha-Ras stimulated *in vivo *metastasis through RalGEF-Ral signaling. RalA and RalB are highly homologous small G proteins belonging to Ras superfamily. They can be activated by Ras-RalGEF signaling pathway and influence cellular growth and survival, motility, vesicular transport and tumor progression in humans and in animal models. Here we first time compared the influence of RalA and RalB on tumorigenic, invasive and metastatic properties of RSV transformed hamster fibroblasts.

**Methods:**

Retroviral vectors encoding activated forms or effector mutants of RalA or RalB proteins were introduced into the low metastatic HET-SR cell line. Tumor growth and spontaneous metastatic activity (SMA) were evaluated on immunocompetent hamsters after subcutaneous injection of cells. The biological properties of cells, including proliferation, clonogenicity, migration and invasion were determined using MTT, wound healing, colony formation and Boyden chamber assays respectively. Protein expression and phosphorylation was detected by Westen blot analysis. Extracellular proteinases activity was assessed by substrate-specific zymography.

**Results:**

We have showed that although both Ral proteins stimulated SMA, RalB was more effective in metastasis stimulation *in vivo *as well as in potentiating of directed movement and invasion *in vitro*. Simultaneous expression of active RalA and RalB didn't give synergetic effect on metastasis formation. RalB activity decreased expression of Caveolin-1, while active RalA stimulated MMP-1 and uPA proteolytic activity, as well as CD24 expression. Both Ral proteins were capable of Cyclin D1 upregulation, JNK1 kinase activation, and stimulation of colony growth and motility. Among three main RalB effectors (RalBP1, exocyst complex and PLD1), PLD1 was essential for RalB-dependent metastasis stimulation.

**Conclusions:**

Presented results are the first data on direct comparison of RalA and RalB impact as well as of RalA/RalB simultaneous expression influence on *in vivo *cell metastatic activity. We showed that RalB activation significantly more than RalA stimulates SMA. This property correlates with the ability of RalB to stimulate *in vitro *invasion and serum directed cell movement. We also found that RalB-PLD1 interaction is necessary for the acquisition of RalB-dependent high metastatic cell phenotype. These findings contribute to the identification of molecular mechanisms of metastasis and tumor progression.

## Background

Metastatic spread of primary tumors is a major determinant of cancer-related death. Metastatic process involves multiple steps including local tumor cells dissemination, survival in blood circulation, arrest in vasculature, extravasation and growth in distant organs and tissues [1]. Investigation of signaling pathways regulating metastasis and associated gene expression changes is an important step for designing therapeutic strategies.

Small G proteins RalA and RalB belong to Ras superfamily [2] and are implicated in tumorigenesis, invasion and metastasis [3-7]. RalA and RalB share 82% amino acid identity [8] and participate in numerous cellular processes such as endocytosis, exocytosis, actin reorganization and cell motility, proliferation and modulation of cancer-associated genes expression (for review see [9]). Like all GTPases Ral proteins cycle between active GTP- and inactive GDP-bound states. Ral can bind to and regulate activity of various proteins including Ral binding protein-1 (RalBP1, RLIP76) [10], phospholipase D1 (PLD1) [11], filamin A [12], exocyst subunits Sec5 and Exo84 [13]. Although RalA and RalB have almost identical effector-binding domains, these two proteins may preferentially utilize different effectors [14].

Ral proteins are activated by RalGEFs, some of which are Ras effectors (i.e. RalGDS, Rgl1, Rgl2). Oncogenic Ras mutations were found in subset of human tumors and cell lines. RalA and RalB were shown to be activated in pancreatic cancers, aggressive malignancies with high frequency of Ras mutations [15]. It was shown that RalA and RalB were necessary for acquisition of aggressive cellular phenotype in diverse models of tumor progression. Ral proteins were capable to stimulate prostate cancer metastasis to bone. Suppression of RalB activity led to decrease of oncogenic Ras-mediated invasion *in vitro *and reduced metastasis after prostate cancer cells intracardiac injection [16]. Ral GTPases also mediated progression of bladder cancer in animal models [17]. However, little emphasis has been made on comparison of individual roles of RalA and RalB and their downstream partners in tumor progression.

Here we compared the influence of constitutively active RalA and RalB expression on tumor progression. We showed that both active Ral proteins enhanced spontaneous metastatic activity (SMA) of HET-SR cells, however RalB was more potent in stimulation of lung colonization, as well as in promotion of cell invasion and directed migration. SMA stimulating effect depends on N-terminus of RalB protein, which is known to be critical for RalB-PLD1 interaction. We also found RalA-dependent increase of extracellular matrix proteinases activity and RalA/B mediated metastasis-associated signaling cascades stimulation.

## Results

### RalB is more potent in metastasis stimulation than RalA

Previously we have shown that introduction of oncogenic Ha-Ras stimulated spontaneous metastatic activity of RSV-transformed hamster embryo fibroblasts (HET-SR cell line) through activation of RalGDS signaling pathway. Overexpression of active RalA also enhanced lung metastasis formation in immunocompetent hamsters [18].

**Figure 1:**
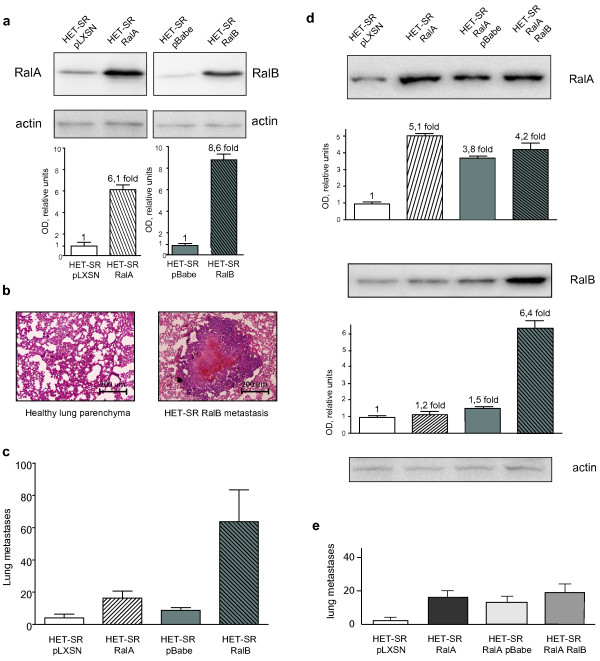
**Ral-dependent stimulation of spontaneous metastatic activity**. (a) Expression of exogenous Ral proteins (RalA-G23V in pLXSN vector (HET-SR-RalA) and RalB-G23V in pBabe vector (HET-SR-RalB)) in HET-SR cell line was confirmed by Western blot analysis using anti RalA or anti-RalB antibodies followed by quantification. (b) Hematoxylin-eosin staining of control hamster lung and metastatic nodule formed by RalB-expressing cells (c.) RalB stimulates spontaneous metastatic activity (SMA) more than RalA. Indicated is number of lung metastases formed two months after subcutaneous injection of cells into adult immunocompetent hamsters; the average for 10 animals is shown with standard error (SE). (d, e) Simultaneous expression of RalA and RalB doesn't give additive effect on SMA. (d) Western blot analysis and quantification of RalA/B expression in HET-SR-RalA-RalB cells obtained by RalB transduction into HET-SR-RalA cells. (e) Graphs represent influence of RalA- and RalA-RalB combined expression on SMA of HET-SR cells.

Here we compared the ability of RalA and RalB to influence metastatic potential and associated properties of tumorigenic low-metastatic HET-SR cell line. For this purpose we generated stable cell lines HET-SR-RalA and HET-SR-RalB expressing active GTP-bound forms of Ral proteins (RalA G23V and RalB G23V) in retroviral vectors (pLXSN and pBabe-puro, respectively). Expression of exogenous Ral proteins was confirmed by Western blot analysis of total cell populations selected on G-418 (HET-SR-RalA) or puromycin (HET-SR-RalB) (Figure 1a).

For evaluation of SMA, 10^4 ^cells of HET-SR-RalA or HET-SR-RalB in parallel with cell lines expressing empty vectors were subcutaneously injected into 10 immunocompetent syngeneic animals. Two months later tumor-bearing hamsters were sacrificed; paraffin-embedded lungs were step-sectioned and stained with hematoxylin-eosin (Figure 1b). Metastatic lesions in lungs were counted microscopically. We found statistically significant increase in metastatic nodules number per animal (in comparison with empty vector-expressing cells) for both cell lines (Figure 1c). RalB-dependent stimulation of SMA was noticeably higher: cells expressing active RalB formed 63.7 ± 19, while HET-SR-RalA formed 20 ± 4 lung nodules per animal in comparison with 7 ± 1.9 and 4 ± 2 nodules formed by cell lines bearing empty vectors, HET-SR-pBabe and HET-SR-pLXSN respectively. Therefore, RalB effect on metastases stimulation was 3 times more than RalA.

### Simultaneous expression of active Ral proteins has no additive effect on metastasis

It was previously shown that RalA and RalB proteins may have non-overlapping or even opposite influence on several cellular properties [4,6,19-21]. At the same time there were no data concerning effect of simultaneous expression of active RalA and RalB on metastasis. To test whether Ral proteins cooperate in SMA stimulation we introduced active RalB in retroviral vector pBabe-puro (or pBabe-puro vector alone as a control) into HET-SR-RalA cells. The expression of RalB in derived HET-SR-RalA-RalB cells was confirmed by western blot analysis (Figure 1d). SMA assay revealed that HET-SR-RalA-RalB cell line formed 16.2 ± 1.3 lung metastatic nodules. This value was comparable to control HET-SR-RalA-pBabe cells (12.3 ± 3.2) and considerably less than HET-SR-RalB cells (63.7 ± 19) (Figure 1e). Therefore we concluded that combined expression of both Ral proteins doesn't give an additional stimulation of SMA compared to the effect of RalB expression alone. This result gives evidence that exogenous RalB expression in the presence of exogenous RalA inhibits original RalB potency of metastasis stimulation.

### Active RalA and RalB similarly modulate cell growth

**Figure 2:**
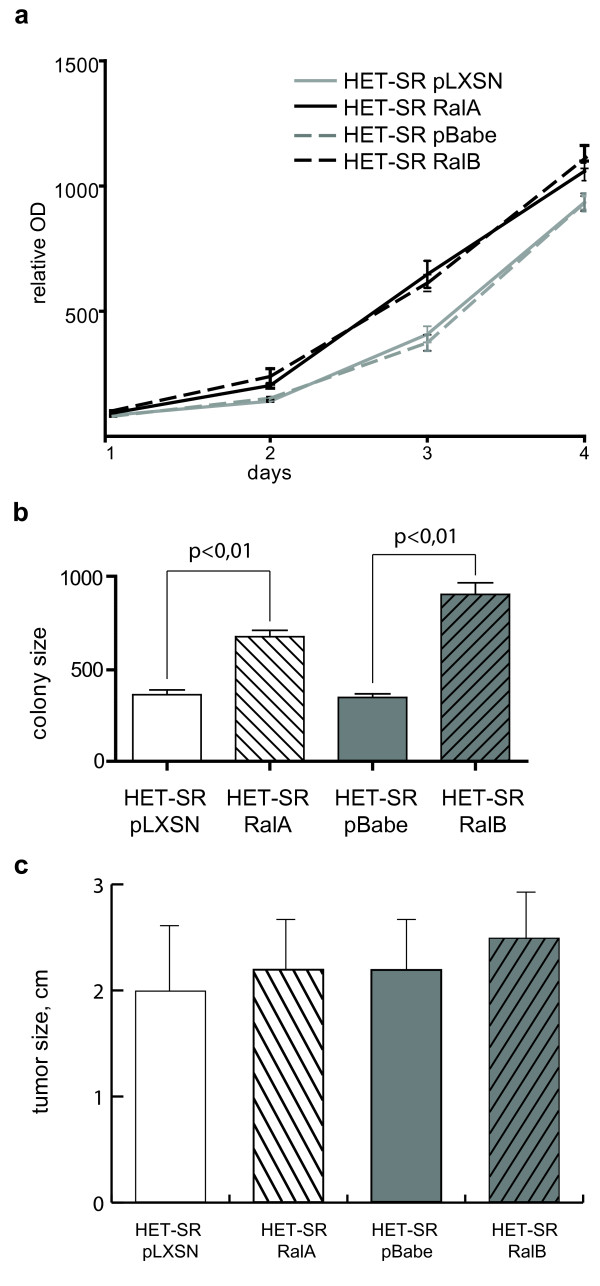
**Effect of active Ral proteins on HET-SR cells growth characteristics**. (a) Ral proteins expression has no significant influence on HET-SR cells proliferation.; OD_595 _absorption in MTT test is shown as average of three independent experiments ± SE. (b) RalB is more active than RalA in stimulation of colony formation in clonogenicity assay. Colonies were measured 6 days after seeding at low cell density (200 cells per 6-cm dish); colony size is shown in arbitrary units as average for three independent experiments ± SE. (c) Neither RalA nor RalB modulate tumorigenicity of HET-SR cells. Indicated is the size of tumors two months after injection as average for 10 animals ± SE.

Aggressive phenotype of transformed cells could be partly defined by their ability to grow in different conditions. To examine the effect of active Ral proteins on cell proliferation, we conducted MTT-test on daily basis for a period of five days. The growth curve of each cell group did not reveal significant effect of RalA or RalB on cell proliferation (Figure 2a). These results suggest that both active Ral proteins do not change proliferation dynamics of studied cells in standard culture conditions.

Next, we studied the influence of Ral proteins on the ability to grow under conditions of rare population (clonogenicity assay) to reveal possible role of these proteins in autocrine or paracrine stimulation of cell growth. We found that both Ral proteins significantly increased the size of colonies after seeding of 200 cells per 6-cm dish (p < 0.01) although RalB was more potent. Thus, RalA expression enlarged colony size 1.9 fold, while RalB gave 2.6 fold increase of this value compared to control cells (Figure 2b). At the same time, no difference in the number of colonies formed by all studied cells was detected (data not shown).

We also tested whether RalA or RalB could stimulate tumor growth *in vivo*. For this 2 × 10^3 ^of control vectors- and RalA- or RalB-expressing HET-SR cells were injected subcutaneously in adult immunocompetent animals. Tumor growth was measured starting from the 14^th ^day after injection (time of measurable tumor appearance). We did not find significant differences neither in growth dynamics (data not shown) nor in tumor size for HET-SR-RalA or HET-SR-RalB cells in comparison with corresponding controls (Figure 2c).

### Ral proteins stimulate cell motility and invasion in vitro

**Figure 3:**
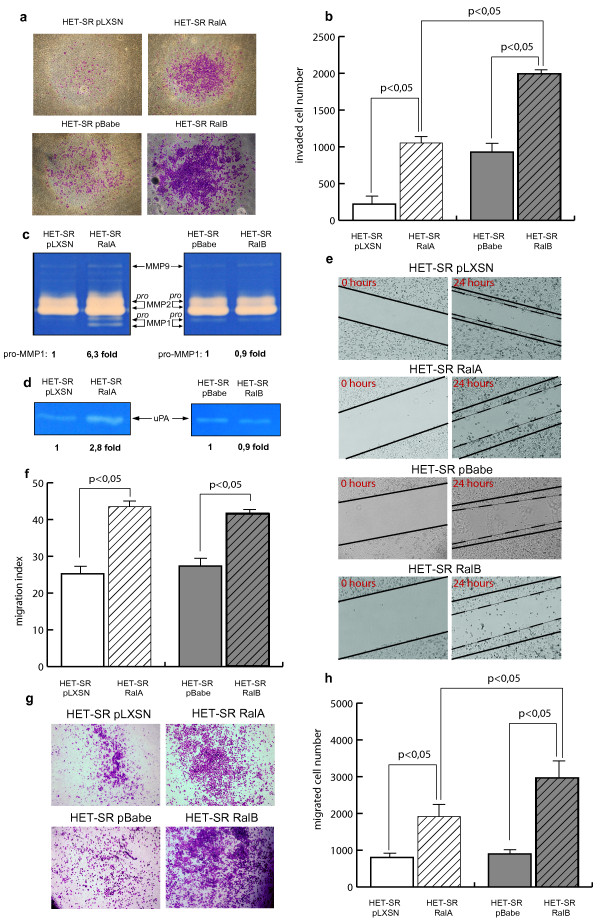
**Migrative and invasive properties of Ral-expressing HET-SR cells**. (a,b) RalB is more active in stimulation of *in vitro *invasion. Invaded cells were stained with crystal violet 18 hours after seeding on Matrigel-coated Boyden chambers; (a) Representative pictures of three independent experiments are shown. (b) Graphs correspond to the number of invaded cells shown as average for three independent experiments ± SE. (c) Gelatin zymography was used to study the activity of matrix metalloproteases (MMPs) in conditioned media. RalA increases MMP-1 proenzyme level (proMMP-1) and drastically stimulates MMP-1 activity, while RalB has no effect on studied gelatinases. (d) RalA opposite to RalB increases activity of urokinase-like plasminogen activator (uPA). uPA activity in conditioned media was revealed by casein-plasminogen zymography. (e,f) Both Ral proteins stimulate cell motility in "wound healing" assay. (e) Representative pictures of wounds at 0 and 24 hours after scratching. (f) Graphs correspond to migration indexes shown as average for three independent experiments ± SE. (g,h) RalB is more potent in stimulation of growth factors-directed migration in transwell assay. (g) Migrated cells were stained with hematoxylin-eosin 18 hours after seeding in uncoated chambers. (h) Graphs correspond to the number of transwell migrated cells shown as average for three independent experiments ± SE.

The *in vitro *invasiveness of Ral-expressing cells towards serum gradient was examined using Matrigel-coated Boyden chambers (Millipore). We found that HET-SR-RalB cells invaded significantly better than HET-SR-RalA cells, although both RalA- and RalB-expressing cells were more active in this test than corresponding control cells (HET-SR-pLXSN and HET-SR-pBabe respectively) (Figure 3a,b). Therefore, both Ral proteins stimulate cell invasion but with different potency. This result is in compliance with Ral-mediated SMA increase of HET-SR cell line.

The ability to penetrate through Matrigel-coated membrane depends on ECM remodeling proteinases activity on the one hand and motility on the other. To study molecular mechanisms of Ral-dependent stimulation of cell invasion we examined the activity of certain extracellular proteinases. We studied matrix metalloproteinases (MMPs) with gelatinase activity and urokinase-like plasminogen activator (uPA). These proteinases are responsible for matrix degradation and thus contribute to tumor progression and metastasis. The secreted proteinases activity was tested in culture media by casein/plasminogen (for uPA) or gelatin (for MMPs) zymographies. MMP-2 was the most active gelatinase secreted by all studied cells. We didn't reveal any difference in MMP-2 and MMP-9 activities between studied cell lines (Figure 3c). RalA-expressing cells demonstrated 6.3-fold increase of MMP-1 proenzyme level compared to the control HET-SR-pLXSN cells. In contrast, MMP-1 activity in HET-SR-RalB cells remained at the same level as in the control HET-SR-pBabe. Moreover, active form of MMP-1 was detected only in RalA expressing cells. Comparison of uPA activity in conditioned media also revealed its significant (2.8-fold) increase in RalA-expressing cells (Figure 3d). Therefore, RalB-associated increase of *in vitro *invasion is unlikely to be caused by differences in studied ECM proteinases activity.

We also tested whether Ral-mediated changes in cell motility could contribute to invasion stimulation. Cells were subjected to *in vitro *"wound healing" assay. As shown in Figures 3e,f, expression of active RalA and RalB lead to similar increase in "wound healing" efficiency (migration indexes for RalA and RalB were 44% and 42%, whereas migration indexes for controls were 25% and 27% respectively).

Results obtained on proteinases activity and wound healing do not explain RalB-mediated increase of invasiveness in Matrigel-coated chambers. This increase could be determined by difference in efficiency of serum-directed migration. We used migration through uncoated porous inserts assay to check this possibility. We found that both RalA- and RalB-overexpressing cells demonstrated higher levels of transwell migration than corresponding control cells. Moreover, RalB was significantly more active than RalA in promoting directed migration (Figure 3g,h).

Thereby, we conclude that the difference in Ral-mediated stimulation of *in vitro *invasion definitely correlated with SMA of studied cells and is most probably determined by the difference in their ability for chemotactic movement.

### Ral proteins regulate CD24, Cav-1, Cyclin D1 and pJNK1 protein expression

**Figure 4:**
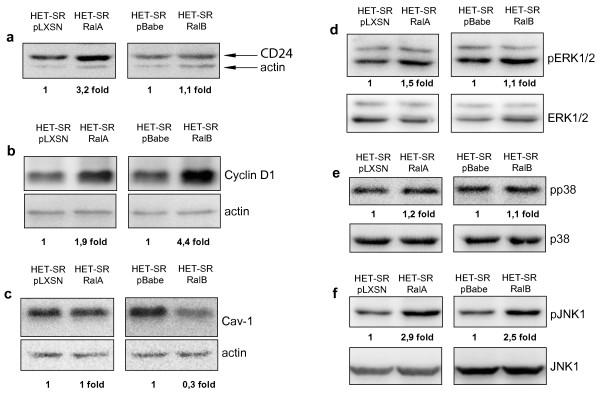
**Influence of RalA and RalB activation on CD24, Cyclin D1, Cav-1 and MAPKs**. (a) RalA upregulates expression of CD24. (b) Both Ral proteins stimulate Cyclin D1 expression. (c) RalB downregulates Caveolin-1 expression. (d, e) Neither of Ral proteins modulate ERK and p38 kinases phosphorylation. (f) JNK1 phosphorylation is equally stimulated in RalA- and RalB-expressing HET-SR cells.

Ral influence on intracellular signaling and protein expression has been intensively investigated. Here we studied some different branches of metastasis-associated signaling pathways, potentially regulated by Ral proteins (Figure 4).

It was previously shown that RalA depletion downregulated cell surface highly-glycosylated protein CD24 expression in bladder cancer cell lines [22]. This molecule may regulate cell survival and proliferation as well as tumor metastasis. CD24 has been proposed to be a marker of pancreatic cancer stem cells and may be associated with unfavorable prognosis [23]. To test whether Ral-mediated increase of SMA is associated with increase of CD24 expression we estimated its level by Western blotting. We revealed that overexpression of active RalA but not RalB upregulated CD24 expression (Figure 4a). This observation suggests that CD24 is unlikely to be a marker of highly metastatic cell phenotype in the studied experimental model.

It was shown that RalB could increase the NF-κB-dependent expression of Cyclin D1 [24]. Cyclin D1 functions as a well-known mitogenic mediator. Recent studies revealed that Cyclin D1 also acts as a motogen and promotes cell migration [25,26]. We tested Cyclin D1 level in cells expressing active RalA and RalB and found that both Ral proteins stimulated expression of this protein in comparison to the control cells (Figure 4b). At the same time RalB-dependent increase of CyclinD1 expression was 2.5 fold higher than in RalA expressing cells. Therefore, elevation of CyclinD1 level in both RalA and RalB expressing cells correlates with stimulation of cell motility. However, RalB influence on CyclinD1 is noticeably stronger than that of RalA, what correlates with more pronounced effect of RalB on SMA.

Caveolin-1 (Cav-1) is a structural protein of caveolae, a special type of lipid rafts that can modulate various proteins activity e.g. Ras, Cyclin D1, Erk1/2, p38 and others [27]. Cav-1 downregulation is associated with aggressiveness of certain tumors and cell lines and it was previously shown that Cav-1 depletion downregulated RalA expression [28]. We found that overexpression of active RalB (but not RalA) in HET-SR cells led to more than 3 fold decrease of Cav-1 (Figure 4c). Therefore Cav-1 downregulation in studied model correlates with aggressive cell phenotype.

We also studied the influence of Ral activation on phosphorylation status of three main MAPK kinases: extracellular signal-regulated kinase (ERK1/2), c-Jun N-terminal kinase (JNK1), and p38 MAPK. We didn't find significant changes neither in ERK1/2 nor in p38 kinase phosphorylation (Figure 4d,e). At the same time, JNK1 was activated both in RalA- and RalB-overexpressing cells (Figure 4f).

### Stimulation of metastasis by active RalB expression depends on RalB-PLD interaction

**Figure 5:**
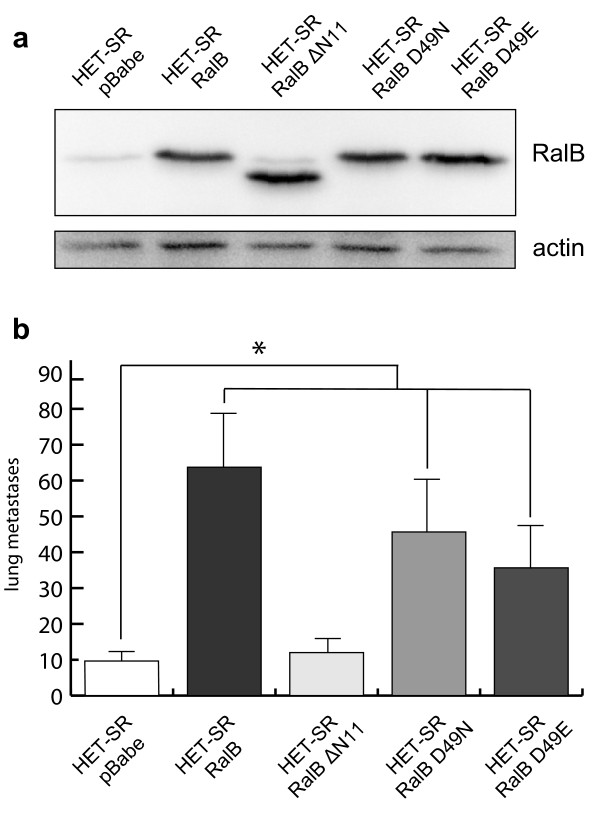
**Among three main RalB effectors, PLD1 is essential for HET-SR-RalB high metastatic phenotype**. (a) Western blot analysis was used to confirm expression of exogenous RalB G23V effector mutants: ΔN11, D49N and D49E (blocking interaction with PLD1, RalBP1 and exocyst complex respectively). (b) SMA test of HET-SR cells expressing active RalB mutants. Activated RalB as well as its effector domain mutants D49N and D49E give statistically significant increase of SMA compared to the control (empty vector expressing cells) (p < 0,05), while deletion of N-terminus leads to abrogation of activated RalB-dependent SMA stimulation.

In order to study RalB-downstream signaling and to reveal RalB partners mainly responsible for metastasis stimulation, we tested the influence of RalB effector mutants on spontaneous metastatic activity of HET-SR cells. Sequences encoding three effector mutants of active RalB: D49N, D49E (effector loop mutants) and ΔN11 (11 N-terminal amino-acids deleted) were cloned into pBabe-puro retroviral vector and stably expressed in HET-SR cells. These three mutations impeded RalB interaction with downstream partners RalBP1 [29], Sec5 and Exo84 exocyst subunits [30] and PLD1 respectively [31]. Expression of RalB mutant proteins was confirmed by Western blot analysis of total cell lysates after selection on puromycin (Figure 5a). *In vivo *analysis of obtained cell lines (Figure 5b) revealed that both D49N and D49E mutations of active RalB significantly (p < 0,05) stimulated SMA in comparison to the control vector. There were no significant differences between SMA of HET-SR-RalB-D49N and HET-SR-RalB-D49E and cells expressing fully active RalB G23V. In contrast, RalB mutant with deletion of 11 N-terminal aminoacids exhibited low level of SMA, comparable to that of control cells. So, blocking of RalB-PLD1 interaction abolishes RalB-dependent metastasis stimulation and suggests phospholipase D1 to be the main RalB effector responsible for acquisition of high metastatic phenotype.

## Discussion

Molecular pathways regulating tumor metastasis to distant organs are still poorly understood. Research in this area is hampered by multiplicity and complexity of steps and factors involved in this process and by the absence of criteria determining the metastatic behavior.

Common *in vivo *models used in different studies are based either on mouse cell lines or on human cultures injected into immunocompromised animals. The model system used here includes HET-SR line that is characterized by high tumorigenicity (minimal subcutaneous inoculation dose - 200 cells) and low lung specific spontaneous metastasis (0-5 metastatic nodules per lung) in syngeneic immunocompetent animals [32-35].

According to mouse models of tumor progression it was previously considered that major Ras-downstream pathways are Raf-MAPK and PI3K signaling cascades. However, further investigation has shown that Ras-RalGEF-Ral signaling in humans dominated in driving Ras mediated progression and metastasis [36,37]. We obtained similar results when investigating Ras-downstream pathways participation in metastasis promotion using HET-SR model [18]. We have shown that RalGEF-Ral branch was the major contributor to the high metastatic phenotype among three main Ha-Ras-dependent signaling cascades.

RalA and RalB constitute a subfamily of proteins within Ras superfamily of small G-proteins. Recent studies indicated that Ral proteins are involved in tumorigenesis and cancer progression. There is still little data regarding comparison of individual contribution of RalA and RalB proteins in stimulation of metastasis.

Here we compared individual impact of RalA and RalB activation on stimulation of tumor growth and metastasis and estimated effect of simultaneous RalA and RalB overexpression on these properties. We also studied effect of Ral proteins on modulation of various cell growth characteristics *in vitro*. For this purpose we generated HET-SR variants with stable expression of constitutively active forms of RalA and RalB. SMA analysis of Ral-expressing HET-SR cells after subcutaneous injection revealed that both RalA and RalB increased SMA but RalB was much more potent in stimulation of lung metastasis.

These results are consistent with the idea that RalA and RalB contribute to different aspects of tumorigenesis. Earlier, it was suggested that RalA was essential for anchorage-independent growth of transformed cells while RalB was responsible for tumor cell-autonomous survival [6,14,19,20]. Data published later proposed the mechanisms of RalB anti-apoptotic action through activation of RalB/TBK1 signaling pathway [38,39]. Moreover, Lim et al. showed that RalA knockdown reduced tumorigenic growth of transformed cells (pancreatic cancer cell lines) while RalB inhibition decreased invasion and experimental metastasis [15]. Noteworthy, in this study metastatic activity was assessed after intravenous injection, thus reflecting later steps of cancer progression, i.e.: ability of cells to survive in circulation and to form secondary focuses. Data presented here give evidence that RalB, significantly more than RalA, stimulates formation of lung metastases after subcutaneous injection.

Further we searched for *in vitro *RalA- and RalB-dependent alterations in cell properties associated with acquisition of aggressive *in vivo *phenotype. Study of growth dynamics didn't show significant changes in proliferation of RalA- and RalB-expressing HET-SR variants. At the same time both RalA and RalB stimulated wound healing as well as Cyclin D1 expression. These results are in concordance with suggested role of Ral proteins in cell motility stimulation [40].

Clonogenicity analysis revealed significant Ral-dependent increase in size but not in number of formed colonies. This effect was more pronounced in RalB than in RalA expressing cells. We can speculate that better adaptation to growth under conditions of rare cell density could reflect abilities to form micrometastases at distant sites.

Results on invasion through Matrigel-coated chambers correlated with SMA of HET-SR-Ral derivatives: Ral-expressing cells were more invasive compared to corresponding controls. At the same time, HET-SR-RalB cells were more aggressive than HET-SR-RalA. This result corresponds with mentioned above data on RalB depletion dependent decrease of invasion and experimental metastasis [15]. Invasion assay combines two processes: proteolytic degradation of matrigel barrier and chemotactic movement on serum gradient. Study of proteolytic activity in conditioned media revealed uPA and MMP-1 stimulation only by active RalA. Therefore, RalB dependent gain of invasion could not be explained by contribution of studied proteinases. At the same time we found that RalB stimulated migration on serum gradient in uncoated chambers more than RalA. This result shows that different invasion capacity demonstrated by studied cells could be defined by chemotactic movement rather than by activity of studied proteases. It could be a result of RalB-dependent changes in growth-factor- or chemokine-receptors regulation or downstream signaling [41].

We also examined some Ral-associated proteins that could serve as potential markers of high metastatic phenotype. Several studies suggest that tumor cells arriving in target organ may roll on activated endothelium before being able to arrest and proliferate [42,43]. It was previously shown that cell surface molecule CD24 expressed on tumor cells can support rolling on P-selectin and thus CD24-P-selectin pathway may be an important element in recruiting tumor cells to target organs [15,44]. CD24 has been shown to be RalA-regulated in a model of bladder cancer-derived cell lines [22]. We confirmed that RalA, but not RalB, upregulated CD24 expression which could contribute to RalA-mediated increase of SMA.

Cav-1 draws attention as a potential platform for signalosome assembly. Alterations of Cav-1 expression in different tumor types are associated with aggressive behavior of cell lines and are proposed to be a prognostic marker for some human malignancies (for review see [27]). It was shown that Cav-1 could modulate activity and expression of several proteins including RalA [27]. We revealed that expression of active RalB, opposite to RalA, lead to decreased Cav-1 expression. Thus, we propose that a feedback loop can exist between Ral proteins and Cav-1, giving additional level of complexity to this branch of signaling.

We also checked whether Ral proteins could influence MAPK signaling cascades in studied model. It was previously shown that Ral proteins could potentiate JNK1 and p38 activation [45-47]. Here we revealed that both RalA and RalB activity induced JNK1 phosphorylation, but we didn't find significant changes in ERK1/2 or p38 activation.

In order to define RalB downstream partners in metastasis stimulation, we used effector loop mutants that abrogated interactions with certain effectors. We studied the SMA-stimulating activity of three RalB effector mutants blocking interactions with PLD1 (ΔN11), RalBP1 (D49N) and exocyst complex (D49E). RalB ΔN11 mutant was the only one incapable to stimulate spontaneous metastasis. So, we propose that interaction with PLD1 is crucial for RalB-dependent lung metastases formation. PLD1 is a well-known second-messenger producer that regulates membrane traffic, cytoskeletal reorganization and cell survival. Its activity was elevated in some human tumors [48]. High levels of PLD1 activity have been shown in T24 bladder and Calu-1 lung cancer cells that harbor mutations in H-Ras and K-Ras, respectively. The PLD1 activity in these cells provided a survival signal that prevented apoptosis in the conditions of serum starvation [49,50]. PLD1 activity can also regulate growth factor receptor endocytosis [51]. We suppose that RalB-PLD1 mediated influence on growth factors signaling is important for RalB-dependent chemotactic movement and stimulation of SMA.

Based on the assumption that RalA and RalB have nonoverlapping functions, we checked the hypothesis that simultaneous RalA and RalB activation may have cumulative effect on SMA. Surprisingly, we found that RalB expression did not strengthen the RalA-mediated increase of SMA. Moreover, simultaneous expression of both Ral proteins resulted in less level of SMA compared to that of RalB alone expressing cells. That might be a result of RalA-PLD interaction which could sequester this effector from RalB. Future studies of RalA and RalB in carcinogenesis and the specificity of their interactions with effectors would hopefully open further opportunities for target drug development.

## Conclusion

Results presented here first time show that RalB activation significantly more than RalA stimulates lung metastasis after subcutaneous injection of transformed cells into immunocompetent animals. This property correlates with the ability of RalB to stimulate in vitro invasion and directed chemotactic cell movement. We also found that among three main RalB effectors (RalBP1, exocyst complex and PLD1) interaction with PLD1 is essential for the acquisition of RalB-dependent high metastatic cell phenotype. Besides, we hope to be the first to study the effect of simultaneous RalA/RalB expression on cell metastatic potential. We also presented here the data concerning effect of RalA and RalB on expression, phosphorylation status and activity of various key proteins known to be involved in tumor progression and metastasis. We suppose that our findings contribute to the identification of molecular mechanisms of metastasis and tumor progression.

## Methods

### Cell cultures and plasmids

GP-293 line was purchased from Clontech; HET-SR (Rous sarcoma virus-transformed hamster embryo fibroblasts) cell line was kindly provided by Dr G.I. Deichman [32], Carcinogenesis Institute, Moscow). All cell lines were maintained in Dulbecco's modified Eagle's medium with 10% fetal bovine serum (FBS; PAA Laboratories) in 37°C and 5% CO_2 _atmosphere. pRK5-RalA-G23V vector and pSRα-RalB-G23V vectors (D49N, D49E, ΔN11) were granted by Dr Jacques Camonis (Transduction du signal et oncogenèse, Institut Curie, France). RalA sequence was cloned into pLXSN retroviral vector by EcoRI and XhoI sites; RalB encoding sequences were cloned in pBabe-puro retroviral vector by BamHI and SalI. All constructs were verified by sequencing.

### Production of stable cell lines

GP-293 cells were cotransfected with retroviral vectors and pVSVG (Clontech) using Lipofectamine 2000 (Invitrogen) according to manufacturer's protocol. 48 and 72 hours after transfection, virus-containing media was applied to 50% confluent HET-SR cells in the presence of 8 mcg/ml Polybrene (Sigma). Infected cells were selected in 1.1 mg/ml G418-containing medium (Calbiochem) for pLXSN-infected cells for 14 days and in 3.5 mcg/ml puromycin-containing medium (Sigma) for pBabe-puro infected cells for 7 days.

### Analysis of tumor growth and spontaneous metastatic activity (SMA) *in vivo*

2 × 10^4 ^cells in 0.5 ml of serum-free media were injected subcutaneously in adult (10 weeks old) Syrian hamsters (*Mesocricetus auratus*). Two months after injection, animals were sacrificed and lungs were collected. Lungs were fixed in alcoholic formalin (10% of formalin and 63% of ethanol). Paraffin-embedded tissues were step-sectioned and stained with hematoxylin-eosin. Metastatic tumor nodules in the lungs were counted microscopically (72 sections per lung per hamster of ten hamsters per group). SMA test for each cell line was performed twice. Tumor growth was hand measured every 7 days.

The animal experimental protocols were approved by the Committee for Ethics of Animal Experimentation and the experiments were conducted in accordance with the Guidelines for Animal Experiments in N.N. Blokhin Cancer Research Center.

### Preparation of conditioned media

4 × 10^5 ^cells were seeded in 6-well plates in full medium. 18 hours later the medium was replaced with 1 ml of serum-free DMEM and 24 hours later the medium was centrifuged 10 minutes at 3000 g. The supernatant was stored at -70°C and used for zymographic analysis.

**Gelatin zymography **was performed using 8% SDS-PAGE gels, containing 0.2% gelatin (AppliChem). Conditioned media samples were mixed 1:1 with zymography sample buffer (0.125 M Tris-HCl pH 6.8; 20% glycerol; 4% SDS; 0.05% Bromophenol blue (Sigma)) and loaded to the gels. After electrophoresis gels were incubated 30 minutes in 2.5% Triton X-100 at room temperature, 30 minutes in collagenase activation buffer (50 mM Tris-HCl, pH 7.4, containing 6.6 mM CaCl_2 _and 200 mM NaCl and 0.2% Brij-35) at room temperature and 4 hours in the same buffer at 37°C. After incubation gels were stained with Coomassie Blue G-250 solution (20% EtOH; 0.08% Coomassie G-250 (Bio-Rad); 1.6% phosphoric acid; 8% ammonium sulfate) overnight. Gelatinases activity was visualized as distinct bands indicating proteolysis of the substrate.

**Casein-plasminogen zymography **was performed in 10% SDS- PAGE gels containing plasminogen (0.04 u/ml, Sigma) and α-casein (2 mg/ml, Fluka). Electrophoretic separation of the conditioned media samples was performed as described for gelatin zymography. Gels were incubated 30 minutes with Triton X-100 (2.5%) at room temperature, 30 minutes in distilled water at room temperature, and 4 hours in uPA activation buffer (25 mM Tris-HCl, pH 7.4, containing 3.3 mM CaCl_2 _and 100 mM NaCl) at 37°C. Caseinolytic bands were visualized after Coomassie Blue G-250 solution staining.

### Western-blot analysis and antibodies

Western blot analysis was proceeded as described previously [18]. Following primary antibodies were used: anti-RalA (Upstate, Millipore), anti-RalB (Upstate, Millipore), anti-Cyclin D1 (Sigma), anti-Caveolin-1 (Sigma); anti-phospho-JNK1 (T183, Abcam), anti-JNK1 (Abcam), anti-phospho-p38 (Y182 and T180, Abcam), anti-p38 (Abcam), anti-ERK1/2 (Cell Signalling), anti-phospho-ERK1/2 (T202 and T204, Cell Signalling), anti-β-actin (Abcam); anti-CD24 (Chemicon). Images of obtained blots were captured using Kodak GelLogic 2200 Imaging system and processed using Kodak Molecular Imaging Software SE ver. 5.0.1.27

### Proliferation assay

For proliferation dynamics analysis 5 × 10^3 ^cells were seeded in triplicate on 96-well tissue culture plates. MTT-analysis was conducted daily. Cell proliferation was analyzed using 3-(4,5-dimethylthiazole-2-yl)-2,5-diphenyl tetrazolium bromide (MTT, Sigma). In brief, 0.5 mcg/ml MTT in media was added to every plate for 1 hour, then cells were lysed using acidic isopropanol and OD data was measured at 595 nm using microplate reader Benchmark Plus, BioRad. Cell doubling time was calculated and graphs were plotted using GraphPad Prizm software, ver. 5.02.

### Wound Healing Assay

3 × 10^5 ^cells were seeded on a 6-well plate and 24 hours later "wounds" were scratched with a 1000-mcl pipette tip, washed with medium and photographed with a digital camera DP71 using inverted microscope Olympus IX-51 (10 × objective lens). Matched pair-marked wound regions were photographed again after 24 hours. The width of the wound at the same position was measured repeatedly by using ImageJ 1.42 I software (6 measures per well and 3 wells per sample, 18 total measurement points per cell line). Migration index was calculated by following formula: % Migration = (the width of initial wound-the width of wound after 24 h) × 100/the width of initial wound.

### *In vitro *invasion assay

Invasive ability of cells was measured with a QCM Cell Invasion Colorimetric Assay (Millipore) according to the manufacturer's protocol. Briefly, cells (2 × 10^5^) in 0.5 ml of serum-free DMEM were seeded into the upper chamber with Matrigel-coated membrane. 0.75 ml of DMEM containing 10% fetal bovine serum was added into the lower chamber. After 18 hours incubating at 37°C, membranes were collected and noninvading cells were removed from the upper surface of the membrane using a cotton swab. Membranes were stained with 0.1% crystal violet, and photographed with digital camera DP71 using inverted microscope Olympus IX-51 with 10 × objective.

### Transwell Migration Assay

Corning Costar Transwell plates (8 μm) were pretreated according to the manufacturer's protocol. Directed motility assay was performed in uncoated chambers in similar conditions, as for *in vitro *invasion assay, but 1 × 10^5 ^cells were seeded in the upper chambers. After incubating (18 hours at 37°C), membranes were collected and noninvaded cells were removed from the upper chamber using cotton swab, stained with 0.1% crystal violet and photographed with digital camera DP71 using inverted microscope Olympus IX-51 with 10 × objective.

### Clonogenicity Assay

2 × 10^2 ^cells were seeded on 6-cm Petri dish. 6 days later formed colonies were fixed with ethanol and stained with crystal violet. Pictures of Petri dishes were taken by compact camera and colonies number and size were measured using ImageJ software.

### Statistical analysis

All cell culture experiments were held in triplicate. Graph data represent the mean ± standard error calculated from indicated number of independent experiments. Differences between two groups were assessed using Mann-Whitney U test. Simultaneous comparison of three or more groups was performed by using Kruskal-Wallis one-way analysis of variance followed Dunns post-test to compare with control group, if it was necessary. Quantification of Western blot data was made by Molecular Imaging Research ver. 5.01 software by Kodak. Results were analyzed and graphs built using GraphPad Prizm ver. 5.02 by GraphPad Software.

## Abbreviations

GEF: guanidine exchange factor; MAPK: mitogen-activated protein (MAP) kinases; MMP: matrix metalloproteinases; PLD1: phospholipase D1; SMA: spontaneous metastatic activity; uPA: urokinase-like plasminogen activator.

## Competing interests

The authors declare that they have no competing interests.

## Authors' contributions

The original plan of the research was devised and written EMT and VAR EMT designed the study, supervised the experiments and discussed the results VAR manufactured cell derivates, performed molecular cloning, cell culture experiments, and western blot analyses. AVK performed gelatin and casein-plasminogen zymographies. AVK performed the MTT assay, wound assay, statistical analysis, performed all calculations and figures and finalized the manuscript. VNA performed tumor growth and SMA analyses, LST performed histological verification and counting of lung metastases. All authors read and approved the manuscript.
